# Unlocking the mystery of heterosis opens the era of intelligent rice breeding

**DOI:** 10.1093/plphys/kiae385

**Published:** 2024-08-08

**Authors:** Zhoulin Gu, Bin Han

**Affiliations:** State Key Laboratory of Plant Molecular Genetics, National Center for Gene Research, CAS Center for Excellence in Molecular Plant Sciences, Institute of Plant Physiology and Ecology, Chinese Academy of Sciences, Shanghai 200233, China; State Key Laboratory of Plant Molecular Genetics, National Center for Gene Research, CAS Center for Excellence in Molecular Plant Sciences, Institute of Plant Physiology and Ecology, Chinese Academy of Sciences, Shanghai 200233, China

## Abstract

Heterosis refers to the phenomenon where the first filial offspring (*F*_1_) from genetically diverse parents displays advantages in growth rate, yield, and adaptability compared with its parents. The exploitation of heterosis in rice breeding has greatly increased the productivity, making a significant contribution to food security in the last half of the century. Conventional hybrid rice breeding highly relies on the breeder's experience on random crossing and comprehensive field selection. This process is time-consuming and labor-intensive. In recent years, rice hybrid breeding has encountered challenges stemming from limited germplasm resource, low breeding efficiency, and high uncertainty, which constrain the progress in yield increase, coupled with difficulties in balancing grain yield, quality, and resistance. Understanding the genetic basis of rice heterosis could lead to significant advancements in breeding concepts and methods. This will fully unleash the advantages of heterosis. In this review, we focus on the research progress of the genetic dissection of crop heterosis and briefly introduce some key advancements in modern intelligent breeding of rice hybrid.

## Introduction

The phenomenon of heterosis is widely observed in animals and plants. It refers to the situation where hybrid offspring, arising from genetically distinct parents, exhibits superior performance in target traits compared with both its parents. Heterosis is extensively applied in agricultural production. Shull (1908) discovered heterosis in maize, and Shull (1946) pioneered the concept of breeding inbred lines to stably produce hybrids with consistent synchronous growth. Subsequently, in the 1930s, the first commercial hybrid maize variety was developed (Duvick 1999). In the 1970s, Yuan Longping and his assistant discovered a naturally pollen-aborted individual in wild rice (*Oryza rufipogon*). This led to the successful development of male sterile lines for rice hybrid, thereby facilitating the application of heterosis in cultivated rice (*Oryza sativa* L.), which is a self-pollinating crop. Hybrid breeding in rice, following semidwarf breeding, marked the second leap in production (Qian et al. 2016), earning it the reputation of the “Second Green Revolution.” The exploration and utilization of heterosis were also carried out in other crops, such as sorghum and potato, to improve their productivity and adaptability (Springer and Stupar 2007; Zhang et al. 2021a; Wu et al. 2023; Paril et al. 2024). Given its economic importance and scientific research interest, researchers have been dedicated to exploring the genetic principles underlying the formation of heterosis, with the ultimate goal of informing breeding practices and improving efficiency. This article reviews the long-standing and recently proposed quantitative genetic explanations for heterosis, summarizes the genetic characteristics of crop heterosis, and further describes the genomic structure shaped by improvement breeding and overviews modern breeding methodologies as well as their development prospects in hybrid rice.

## Genetic basis of heterosis

### The classical hypothesis of heterosis

**Figure 1.**
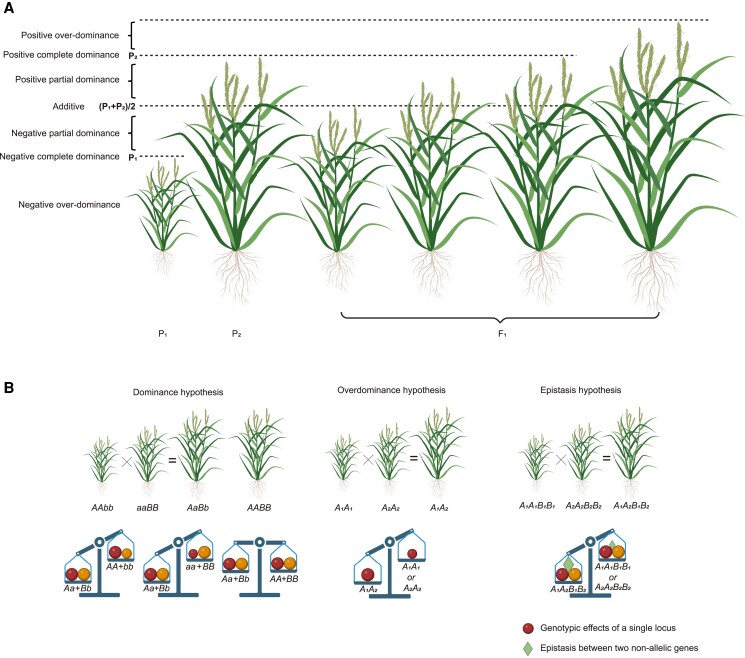
Degree of dominance and 3 classical genetic hypotheses underlying heterosis. **A)** The schematic diagram demonstrates the degree of dominance by rice plant height. *P*_1_ and *P*_2_ represent the genotypic values of 2 homozygous genotypes, and *F*_1_ represents the genotypic value of the heterozygous genotype. When the genotypic value of the heterozygous genotype equals the mean of 2 homozygous genotypes, the heterozygote represents an additive effect; when the genotypic value of the heterozygous genotype is greater than the mean of 2 homozygous genotypes but less than that of the homozygous genotype with an increasing effect (*P*_2_ here), the heterozygote is considered to be positive partial dominant, whereas if it is less than the mean of 2 homozygous genotypes but greater than that of the homozygous genotype with a decreasing effect (*P*_1_ here), the heterozygote shows a negative partial-dominance effect; when the genotypic value of the heterozygous genotype equals that of the homozygous genotype with an increasing effect, the heterozygote represents a positive complete-dominance effect, whereas if it equals that of the homozygous genotype with a decreasing effect, the heterozygote is considered to be negative complete dominant; when the genotypic value of the heterozygous genotype is greater than that of the homozygous genotype with an increasing effect, the heterozygote represents a positive overdominance effect, whereas if it is lower than that of the homozygous genotype with a decreasing effect, the heterozygote is regarded to have a negative overdominance effect. **B)** The schematic diagram depicts dominance, overdominance, and epistasis hypotheses with plant heights (upper) and genotypic effects (lower). The circle represents the genotypic effect of a single locus, and the triangle represents the epistasis effect between 2 nonallelic loci. Created with BioRender.com.

The 3 classical genetic hypotheses underlying heterosis are dominance, overdominance, and epistasis (Fig. 1). The *dominance hypothesis*, also known as the dominant complementation hypothesis, posits that the aggregation of dominant alleles at multiple loci from both parents gives rise to heterosis (Jones 1917). According to this hypothesis, there are deleterious or unfavorable allelic variations at different loci in inbred lines, manifesting as recessive alleles. Hybridization combines multiple dominant alleles from both parental lines in hybrids and effectively conceals the phenotypic impact of recessive alleles. Underpinned by this hypothesis, an inbred line aggregating advantageous loci from both parental lines would theoretically exhibit performance on a par with the hybrid; however, the creation of such inbred lines is laborious and time-consuming (Schnable and Springer 2013). Thus, hybrid breeding remains the optimal approach for swiftly combining favorable loci from both parents. The *overdominance hypothesis* proposes that the heterozygous genotype exhibits superior performance compared with both homozygous genotypes (East 1936; Shull 1948), and different alleles do not exhibit superiority or inferiority, or different alleles exhibit superiority at different growth stages. Consequently, based on this hypothesis, hybrid breeding cannot be substituted by inbred line breeding. It is noteworthy that, in the scenario where 2 tightly linked loci possess genotypes with repulsion-phase phenotypic impact and exhibit dominant effects, and the parents are complementary at these 2 loci, the hybrid would display a *pseudo*-overdominance effect. The *epistasis hypothesis* emphasizes the interplay among nonallelic genes (Minvielle 1987). In the context of quantitative traits, where multiple genes collectively regulate the target traits, this hypothesis postulates that the effect of a particular gene on the target traits can be influenced by one or more other genes.

Several studies have validated that the 3 hypotheses are certainly not exclusive. A study of yield heterosis in maize proposes that additive and dominant QTLs (quantitative trait loci) predominate in vegetative growth stage, while epistatic QTLs are activated and become dominant as plants enter the floral transition period (Xiao et al. 2021). When exploring the genetic basis of inter-subspecific heterosis in rice, researchers find that both dominant and overdominant QTLs play a role in yield heterosis. Notably, dominant QTLs play more substantial roles due to their larger phenotypic variance explained (Huang et al. 2016; Gu et al. 2023). Also, an analysis involving an “immortalized *F*_2_” population from the elite hybrid variety Shanyou63 indicates that overdominance/*pseudo*-overdominance is pivotal in the heterosis of yield, grain number per panicle, and grain weight, while dominance–dominance interaction is found to be vital for heterosis in panicle number per plant and grain weight and also plays roles in yield and grain number heterosis (Zhou et al. 2012a).

Although the genetic framework behind heterosis is complex and can vary across or within species, several common genetic characteristics of crop heterosis have been revealed as investigations delve deeper into this field.

### Genetic features of crop heterosis

#### Trait specificity

Trait specificity is manifested in 2 dimensions: Firstly, *the magnitude of heterosis is trait-specific*. Certain traits, such as plant height in rice, readily exhibit hybrid vigor, while others, such as grain quality in rice, are less likely to display any hybrid advantage (Gu et al. 2023). In terms of grain yield per plant as well as yield components, variations were observed in the degree of middle-parent heterosis among them (Xie et al. 2022). Secondly, *the genetic components to heterosis are trait-specific* (Zhou et al. 2012a). The genetic effects of allelic interactions include additive and partial-, complete-, and overdominance effects, and the epistasis effect caused by nonadditive combinations between nonallelic loci (here referring only to 2-site interactions) includes additive by additive, additive by dominance, and dominance by dominance effects, depending on the nature of the interaction. It has been reported that most of the QTLs controlling grain quality–related traits in rice show negative dominance effects, which indicates that the performance of heterozygous genotypes is not as good as middle-parent value. This finding is consistent with the observation that heterosis is uncommon for grain quality in *indica*–*indica* hybrid rice (Gu et al. 2023), while most of the QTLs identified for grain yield-related traits exhibit positive partial-dominance effects, and cumulative effects of multiple loci give rise to better parent heterosis (Huang et al. 2016). Different quantitative traits are controlled by different sets of QTLs with varying genetic effects, which might be responsible for the trait specificity of heterosis.

#### Quantifiability

**Figure 2.**
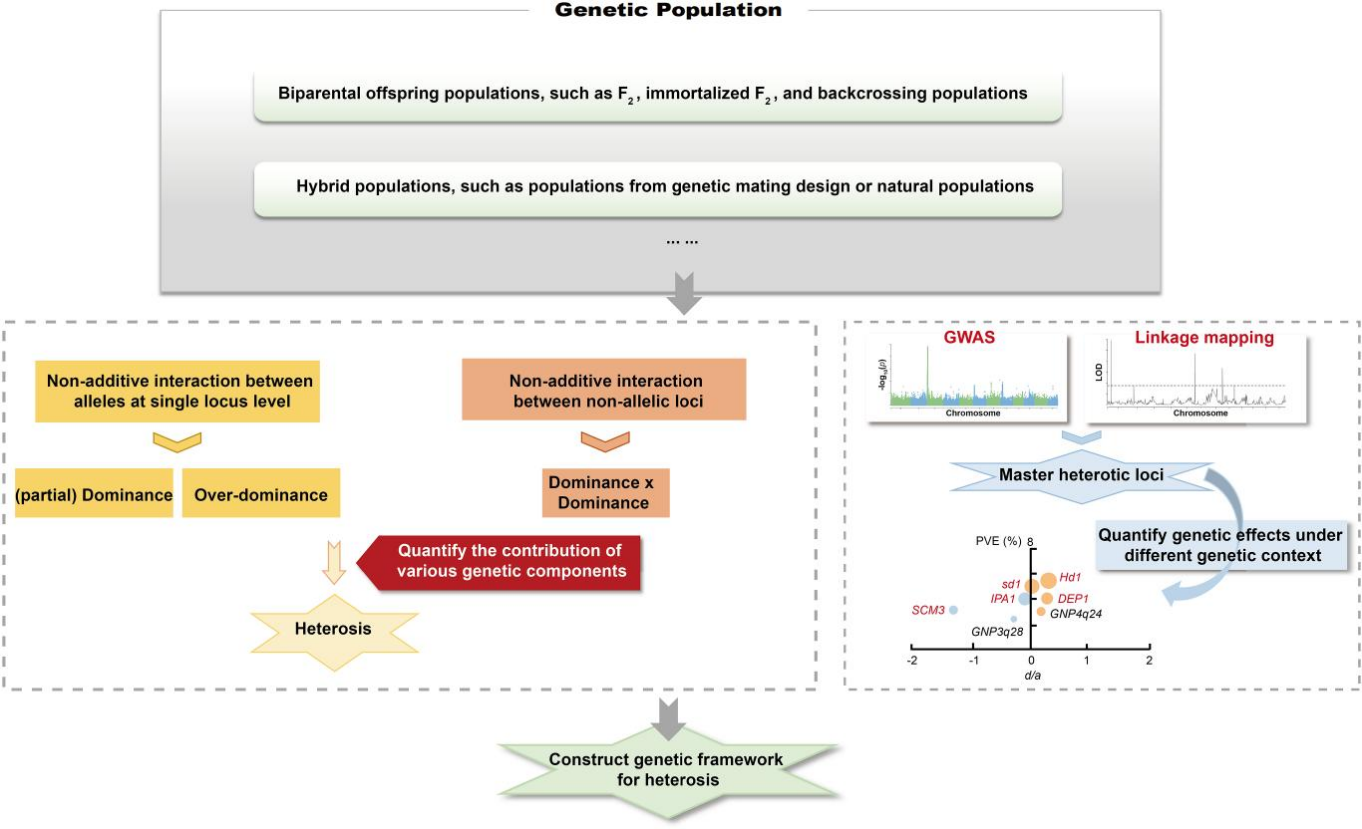
Quantitatively assessing the contribution of nonadditive genetic components to heterosis (left), and further elucidating the genetic architecture of rice heterosis. Identifying and evaluating the genetic effects of master loci underlying heterosis under various genetic backgrounds (right), and further clarifying appropriate targets for molecular design breeding.

Multiple genes contribute to heterosis, and varied magnitudes of dominance effects (partial-, complete-, and overdominance) are observed among these genes (Schnable and Springer 2013; Huang et al. 2016; Boeven et al. 2020; Gu et al. 2023). The findings suggest that heterosis is regulated in a *dosage-sensitive* way (Birchler 2016). Dosage sensitivity refers to the phenomenon in which the quantity of protein produced by the genes affects the plant's characteristics to some degree. Dosage-sensitive modifiers, such as transcription factors, chromatin proteins, or members of signal transduction cascades (Birchler and Veitia 2010), are identified as key regulators of heterosis. For example, the floral regulator *Hd3a* (Os06g0157700) and the transcription factors *Ghd7* (Os07g0261200) and *Ghd8* (Os08g0174500) are reported as master contributors to yield heterosis in rice (Huang et al. 2016; Li et al. 2016; Wang et al. 2022). As emphasized by East (1936): “the problem of heterosis is the problem of the inheritance of quantitative characters,” it is reasonable to construct a quantitative framework involving multiple dosage-sensitive components to explain heterosis. Thus, to gain a comprehensive understanding of the genetic basis of heterosis, it is essential not only to identify the regulators involved, but also to quantitatively assess their genetic effects, including the magnitude of dominance effect and phenotypic variance contribution. In rice hybrids, a quantitative analysis has been performed to elucidate the genetic basis of inter-subspecific heterosis and reveals that QTLs with positive partial-dominance effects play a leading role in yield heterosis due to higher phenotypic variance contribution compared with overdominant QTLs (Gu et al. 2023). Follow-up work, based on carefully designed genetic population in rice and other crops, is essential to further quantitatively dissect the genetic components contributing to heterosis, encompassing genetic effects from both allelic and nonallelic interaction (Fig. 2).

#### Context dependence

A previous study in wheat highlights a positive correlation between grain yield heterosis and genetic divergence of heterotic loci—the QTLs contributing to heterosis (Boeven et al. 2020). In maize, a significant positive correlation is observed between the number of structural variations (SVs) presenting in both parental lines and better parent heterosis of grain yield per plant, while the number of single nucleotide polymorphisms and small insertion and deletion in the syntenic regions only show weak correlation with heterosis (Wang et al. 2023). And this work also documents 2 heterotic loci located in SVs of biparental lines contribute to yield heterosis. The number of superior heterotic loci strongly correlates with grain yield heterosis in rice and biomass heterosis in Arabidopsis, and the genome-wide heterozygosity of hybrids makes little contribution to heterosis (Huang et al. 2015; Yang et al. 2017). These observations indicate that the number of accumulated heterotic loci (dominance complementation) or the number of heterozygous heterotic loci (overdominance) rather than genome-wide heterozygosity determines the magnitude of heterosis in crops. Rice hybrids from 2-line and 3-line breeding systems exploit different sets of heterotic loci (Huang et al. 2016; Lv et al. 2020). Moreover, different sets of genes regulate various growth periods; consequently, the genetic composition of heterosis also shifts in accordance with the changing growth periods. It has been reported in maize hybrids that several major-effect additive and dominant QTLs control heterosis in early vegetative growth phase, while in reproductive stage, widespread and minor-effect epistatic QTLs contribute to heterosis (Xiao et al. 2021). Nonadditive expression, which is associated with heterosis, is more variable under different genetic background and across tissues in rice hybrids (Xie et al. 2022). And 2 different alleles of the *TAC1* (Os09g0529300) gene are, respectively, possessed by maternal and paternal parents and dominant in different tissues and growth periods, thus the functions of the 2 alleles are fully exerted, and the gene represents an overdominance effect in hybrids (Shao et al. 2019). Therefore, the genetic effects of heterotic loci are contingent on the genetic context. It is essential to systematically quantify the genetic effects of master contributors to heterosis across diverse genetic backgrounds and tissues. This will enable future rational utilization of such loci in breeding through molecular design (Fig. 2).

### Beyond 3 classical heterosis models

Based on the dosage-sensitive and context-dependent characteristics, the *gene balance hypothesis* was firstly proposed by Birchler et al. to explain gene regulatory mechanisms with regard to the effect on quantitative trait, polyploidization events, and aneuploidy, and was subsequently applied for heterosis (Birchler and Veitia 2007, 2010; Birchler et al. 2010). According to the hypothesis, heterosis is largely affected by the kinetics and assembly modes of multisubunit protein complexes, which include various regulatory components functioning in hierarchical networks. The hypothesis emphasizes the multigenic nature of heterosis, and determinants of heterosis exhibit some degree of dosage sensitivity. The determinants or regulatory genes exhibit a more optimal stoichiometric balance in hybrids compared with the parental lines. Then, the *unifying theory* proposed by Goff (2011) explains the molecular mechanisms of multigenic heterosis from the perspective of *protein synthesis and metabolism*. The theory emphasizes that higher energy-use efficiency and faster cell cycle progression in hybrids result from the lower rate of protein metabolism. Hybrids acquire a pair of alleles from both parents, one encoding a stable or efficient protein and another encoding an unstable or inefficient protein. The degradation of inefficient protein is energy-consuming. Hybrids could detect and downregulate transcripts encoding inefficient proteins, thereby reduce the production of defective proteins, and conserve on more energy for cell division and growth. And the theory could well elucidate the dominance and overdominance heterosis models: In the dominance hypothesis, the deleterious recessive alleles encode the unstable or inefficient proteins, and crossing rapidly aggregates alleles encoding the stable protein from both parents and downregulates unstable alleles; in the overdominance hypothesis, 2 different alleles from both parents are stable, and they function in different tissues, growth periods or environmental conditions, and crossing brings them together and provides hybrid higher activity and stability of the protein over multiple tissues, growth stages, and conditions. Recently, the *HoIIB model* was proposed by Xie et al. (2022) to explain the core molecular mechanism of heterosis. The model argues that under insufficient background the function of homozygotes could not be activated in parents, while full function of one allele could be activated in hybrids; thus, heterozygotes exhibit relative advantages over homozygotes. The model elucidates that homozygous function constraints in an insufficient background are the core molecular mechanism underlying heterosis.

## Exploitation of heterosis in hybrid rice

### Heterotic group in hybrid rice

As rice is a strictly self-pollinated crop, production of sufficient quantities of hybrid seeds is very difficult and time-consuming, which limited the application of heterosis in rice until the discovery of male sterility resources. The discovery of a naturally pollen-aborted individual in wild rice promoted the commercial development of hybrid rice breeding in 1970s. Subsequently, to broaden the genetic diversity of hybrid rice and simplify the hybrid production system, researchers have sought to exploit new types of male sterility resources (Cheng et al. 2007). Based on cytoplasmic male sterility (CMS), the 3-line breeding system is established to efficiently produce hybrid seed, and the system requires a CMS line, a restorer line, and a maintainer line (Chen and Liu 2014; Kim and Zhang 2018). According to the lineage of CMS lines, the major types of CMS lines and corresponding commercially cultivated 3-line hybrid include WA (wild abortive), ID (Indonesia), G (Gambiaka), D (Dissi), DA (Dwarf Abortive), K, Y, Maxie, BT (Chinsurah Boro II), Dian, HL (Honglian), and other types (Cheng et al. 2007; Luan et al. 2013). Based on the environment-sensitive genic male sterility (EGMS), 2-line breeding system is established and contains the EGMS line and restorer line (Chen et al. 2011; Chen and Liu 2014; Fan and Zhang 2018). The pollen fertility of the EGMS line is reversible and dependent on the environmental conditions. This characteristic allows the EGMS line to serve as both a male sterile line to produce hybrid seeds and a maintainer line to self-propagate. According to the environmental cues causing fertility reversibility, the 2 major types of commercially used EGMS lines are photoperiod- and thermos-sensitive genic male sterility lines (Ding et al. 2012; Zhou et al. 2012b, 2014; Fan et al. 2016). Moreover, humidity-sensitive genic male sterility is also reported, although it has not yet been put into widespread commercial use (Xue et al. 2018; Chen et al. 2020).

**Figure 3.**
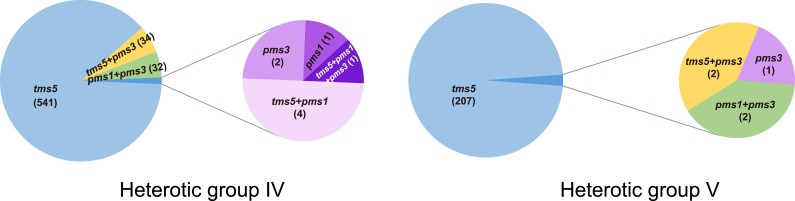
The EGMS-causative genes exploited by rice hybrid heterotic groups IV and V.

Efficient selection of rice hybrid combinations requires the classification of male sterile resources and a comprehensive understanding of their genetic characteristics. Cytoplasm is maternally inherited, recording maternal genealogy information. Additionally, the mitochondrial genome carries the causative gene for CMS. Based on the information provided by these genes, we could easily infer the breeding systems and the genetic basis of fertility recovery and sterility maintenance utilized in hybrid seed production. Utilizing the genomic information of both cytoplasm and nucleus, male sterile lines and corresponding hybrids have been categorized into 5 groups (Gu et al. 2021, 2023). Three distinct heterotic groups are identified within 3-line breeding system, and they, respectively, leverage restoration–maintenance systems governed by the male sterility-causative genes *WA352c*, *orf79*, and *orfH79* (Yi et al. 2002; Luo et al. 2013; Wang et al. 2013; Kazama et al. 2016). Within the 2-line breeding system, 2 heterotic groups with *japonica-*genealogy (heterotic group IV) or *indica*-genealogy (heterotic group Ⅴ) are delineated (Table 1), and the EGMS-causative gene *tms5* is widely used in both heterotic groups (Fig. 3). Although majority *japonica*-genealogy hybrids are *indica*–*indica* combinations, they have higher *japonica*-introgression level compared with other *indica*–*indica* rice hybrids (Gu et al. 2023). Among the 5 heterotic groups, differentiated genomic sequence and distinct crossing patterns are observed. The sequence divergence might result from differentiated founder germplasms among heterotic groups and genetic drag brought by the loci associated with male sterility maintenance and recovery.

**Table 1.** The genetic attributes of 5 heterotic groups in rice hybrid

Heterotic group	Subspecies^a^	Breeding system	Types of male sterile lines	Male sterility–causative gene	Proportion (%)^b^
I	*indica*–*indica*	3-line	WA, ID, G, K, D, DA, Maxie, Y, others	*WA352c*	62.05
II	*indica*–*japonica*; *japonica*–*japonica*	3-line	BT, Dian, others	*orf79*	3.74
III	*indica*–*indica*	3-line	HL, others	*orfH79*	0.64
IV	*indica*–*indica*	2-line	EGMS	*tms5, pms1, pms3*	24.96
V	*indica*–*indica*	2-line	EGMS	*tms5, pms1, pms3*	8.61

^a^The subspecies of the majority (≥95%) materials.

^b^The data are from Gu et al. (2023).

### Genetic improvement during hybrid rice breeding

**Figure 4.**
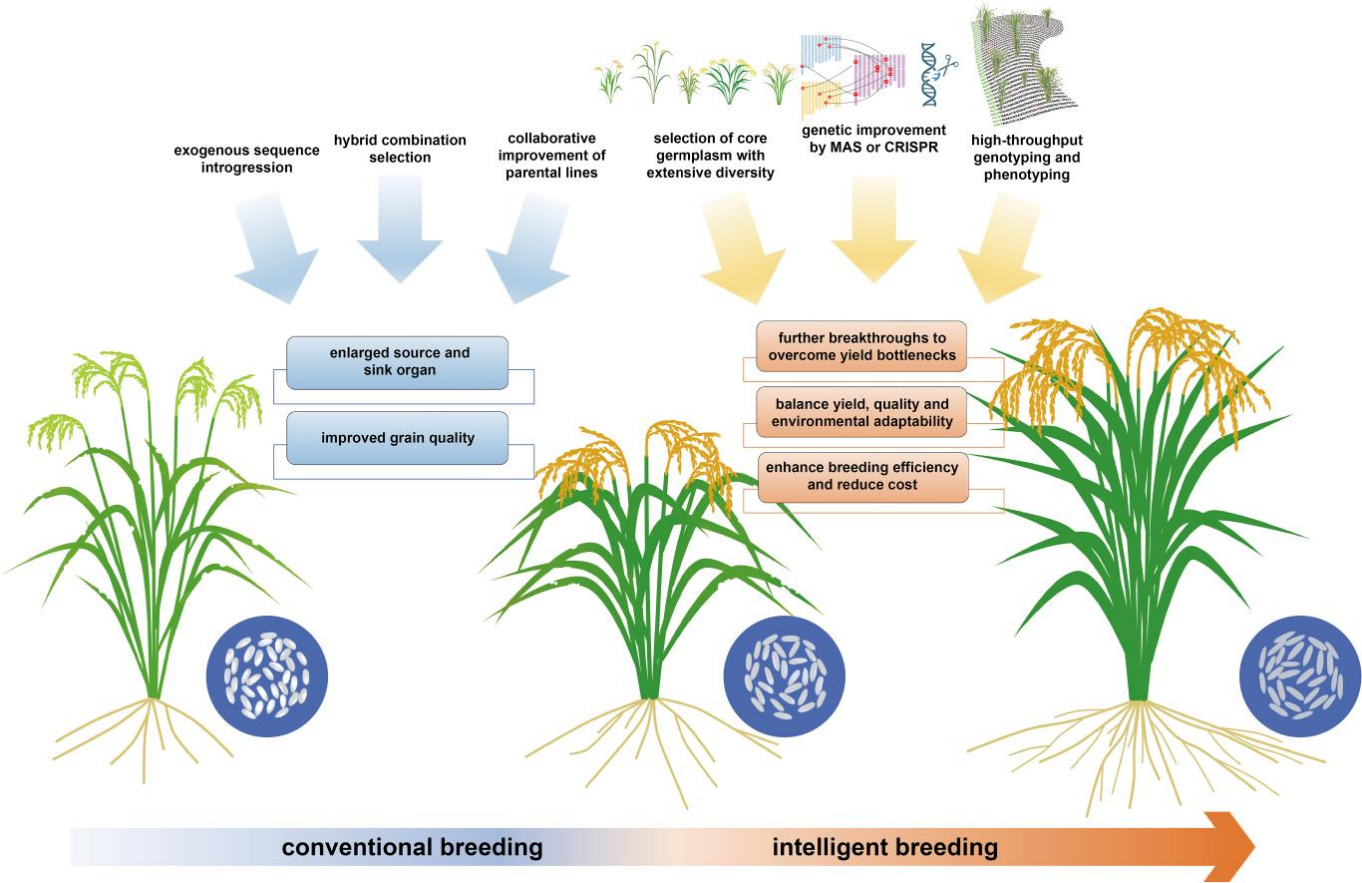
The traditional practices for improvement breeding and the modern methodologies for intelligent breeding.

Hybrid breeding has progressed over the past half century, resulting in the development of numerous elite hybrid varieties. Improvement breeding in rice hybrids has significantly increased grain yield (Cheng et al. 2007; Li 2020), with enlarged source (flag leaf) and sink organ (grain numbers); moreover, the grain appearance and cooking quality have also been greatly improved (Fig. 4; Gu et al. 2023). Among hybrid varieties and parental lines from the entire span of breeding history, their genomes record the genetic footprints of improvement breeding. Thus, comprehensively examining and comparing a large set of hybrid varieties and parental lines can provide insight into the genetic basis underlying improvement breeding: (i) introgression of exogenous sequences from other rice subpopulations shapes the genomic structure. Among the 5 heterotic groups in rice hybrids, the heterotic group IV has a higher *japonica*-introgression level compared with other *indica*–*indica* heterotic groups (Gu et al. 2023). Peiai64S, the typical male sterile line for heterotic group IV, originates from a cross involving tropical *japonica* and *indica* germplasms. Peiai64S is an *indica*-inclined variety, with approximately 11% the *japonica* genetic component in its genome. Moreover, male and female parents also possess different levels of introgressions from diverse rice subpopulations, including *indica* (*Ind I* sequence is introgressed into *Ind II* genome), *aus*, and *japonica* (Lin et al. 2020). The introgression events also brought breeding-favorable alleles or shaped the heterotic loci, which subsequently underwent divergent selection driven by breeding preference or environmental adaptation throughout the breeding process, and in turn shaped the genomic structure of rice hybrid and parental lines. (ii) Harnessing new male sterility resources and innovating germplasm have broadened genetic diversity during improvement breeding. After the 20th century, there was a boom in 2-line hybrid varieties, which are genetically differentiated with 3-line hybrid varieties (Huang et al. 2016; Lv et al. 2020; Gu et al. 2023). The wide cultivation of 2-line hybrids has increased the genetic diversity of rice hybrids. (iii) Hybrid combination selection and collaborative parental improvement have pyramided breeding-favorable alleles in rice hybrids. Throughout the course of hybrid rice breeding, there is a noticeable accumulation of breeding-favorable alleles. Optimization of hybrid combination to take advantage of genetic complementation, along with the collaborative incorporation of breeding-favorable alleles into both parents at loci representing negative dominance effects, stands as cornerstones in improvement breeding (Gu et al. 2023). The improvement in grain quality-associated traits is representative. The majority master loci governing grain quality exhibit negative dominance effects, where heterozygotes perform below the mid-parent value, leading to poor complementarity at these loci. This observation is consistent with the inherent impression that the grain appearance and cooking quality of hybrid rice are poor. During the improvement breeding, both parents are furnished with the breeding-favorable alleles at the major loci controlling grain quality, guaranteeing the hybrid acquire the homozygous beneficial genotypes. For example, due to the negative dominance effect for heterozygous genotype of the *Waxy* gene (Os06g0133000), more than 70% rice hybrids bred after 2010 possess the homozygous advantageous genotypes, whereas only 27% hybrids generated before 2010 hold such genotype.

A comprehensive grasp of genetic basis underlying heterosis and improvement breeding could, in turn, catalyze the innovation of breeding concepts and techniques, thereby enhance breeding efficiency, and maximize exploration of crop heterosis to its fullest extent.

### Modern methodology for hybrid rice improvement breeding

Traditional hybrid breeding relies on random crossing and field selection, being laborious and time-consuming. The development of sequencing technology and the reduction in sequencing costs have led to a surge in sequencing data, the establishment of high-throughput genotyping techniques, an increasing identification of functional variants, and a clearer understanding of the genetic basis underlying complex traits. These advancements further facilitate intelligent breeding, also known as Breeding 4.0 (Wallace et al. 2018). In contrast to conventional breeding, intelligent breeding leverages big data and deeply integrates biotechnology with digital technologies to achieve efficient and precise breeding. Based on the entire process of breeding practice, we overview the technological advancements propelling the progress of intelligent breeding (Fig. 4).

#### Selection of core germplasm

For parental line improvement, a common scheme involves using an elite cultivar as the receptor or backbone material and selecting donor materials to provide target loci for the receptor material. Some elite backbone germplasms, such as Zhenshan97A, are widely employed in traditional breeding of parental lines, resulting in relatively limited genetic diversity. In order to facilitate selecting core collections as backbone lines and foster the utilization of diverse germplasm, a wealth of data resource, including phenotype, genotype, and phylogenetic information of rice cultivars and hybrids, is being generated (Huang et al. 2015; Wang et al. 2018; Lv et al. 2020; Gu et al. 2023). Moreover, the RiceNavi platform integrates genotype of 404 diverse rice accessions and 348 causative quantitative trait nucleotides (QTNs). The platform is developed to select appropriate donor samples and optimize breeding routes for QTN pyramiding and genetic improvement (Wei et al. 2021). In traditional breeding, elite hybrid crosses are identified by large-scale testcrosses, which are time- and labor-intensive; thus, it is impossible to test all possible combinations. This leads to some potentially elite combinations being overlooked. Genomic selection (GS) serves as a powerful tool for rapidly screening superior genotypes (Crossa et al. 2017). GS utilizes genome-wide markers to predict the genomic estimated breeding value (GEBV) of target individuals, and it can calculate the GEBV for hundreds and thousands of samples in a short time. Based on predicting results, GS aids in efficient selection of appropriate materials or combinations for further genetic improvement and assists in design of breeding programs. Leveraging a comprehensive dataset from rice hybrid varieties and segregating populations, GS methods for optimizing hybrid combination have advanced over the past decade and continue to evolve (Xu et al. 2014; Cui et al. 2020b; Chen et al. 2023a; Gu et al. 2023). Although GS has shown its applicability in rice hybrid breeding, before the widespread application of GS technology, we still need to consider and address the following issues: How to efficiently and accurately integrate phenotype and genotype datasets from different sources? How to construct a GS model applicable to various rice hybrid ecotypes and environmental conditions? How to further simplify the procedure of GS, making it user-friendly for those lacking bioinformatics expertise?

#### Strategy for genetic improvement in backbone materials

Advancements in functional genomics in rice have significantly enriched our understanding of genes controlling important agronomical traits, hastening the progress of the rational design by marker-assisted backcross (MABC) and marker-assisted gene pyramiding (MAGP) (Qian et al. 2016). Based on the knowledge of genes regulating grain yield and quality, the rational design approach successfully pyramided the beneficial alleles in the backbone line and achieved the desired characteristics (Zeng et al. 2017). The rational design strategy can effectively improve multiple quantitative complex traits concurrently and significantly shorten the breeding period to only 5 yr. Based on the genome-wide genotype of backbone accessions and the knowledge of target loci for genetic improvement, the RiceNavi system could design a breeding route, which picks candidate donors, simulates the appropriate number of backcross, and selects optimal individuals in backcrossing population for the next generation (Wei et al. 2021). It has been proven to be significantly faster and more precise than conventional breeding in the application of genetic improvement. Moreover, several strategies have also been proposed in instances where information regarding target loci for genetic improvement is lacking. Such strategies usually conduct genetic dissection of targeting traits prior to genetic improvement. A restorer improvement strategy has been proposed based on the performance of *F*_1_ generation and information of QTLs mapped in *F*_2_ generation (Wang et al. 2019). Given that some loci regulating yield-related traits represent partial-dominance effects, where heterozygotes do not perform as well as the advantageous homozygotes (Huang et al. 2016), the strategy introduces maternal-origin superior alleles into the restorer line and aims to further enhance the performance of hybrids. Firstly, backbone germplasm is testcrossed with a series of other germplasms; subsequently, *F*_1_ lines are compared with the backbone germplasm and the *F*_1_s with superior performance in target traits are selected to generate a *F*_2_ population. Using a Composite Interval Mapping or GradedPool-Seq mapping methods, causative loci are located. Finally, genetic improvement backbone lines are attained through MABC and MAGP. Further, a strategy of breeding by selective introgression (BBSI) has been proposed (Zhang et al. 2021b). BBSI is implemented by development of introgression lines under elite genetic background, genetic dissection of complex quantitative traits, and conduction of genetic improvement through MABC and MAGP. Furthermore, tapping heterosis by Clustered Regularly Interspaced Short Palindromic Repeats (CRISPR)-based genome editing technology is on the horizon. CRISPR enables the rapid introduction of one or multiple desired alleles by editing target loci, creating transgene-free materials (Li et al. 2019; Zhou et al. 2022; Wei et al. 2024). In contrast to conventional introgression method, CRISPR is time- and labor-saving and is unaffected by genetic linkage drag. Additionally, editing the loci conferring hybrid incompatibility in inter-subspecific combinations could break down the reproductive barrier without affecting other agronomical traits (Xie et al. 2017), thereby holding the potential to promote the utilization of strong inter-subspecific heterosis. And novel beneficial alleles have also been created by CRISPR. For example, *IPA1* and *SCM3*/*OsTB1* have pleiotropic effects, and the allele increases tiller numbers but decreases grain number per panicle. By CRISPR, new alleles are created to strike a balance or decouple the tradeoff between panicle number and size and hold great potential to break the bottlenecks of grain yield (Cui et al. 2020a; Song et al. 2022). CRISPR is also applied to create environment-insensitive genic male sterile resources, which are crucial for establishment of a “third-generation” hybrid rice breeding system (Chang et al. 2016; Wang and Deng 2018; Chen et al. 2023b). When compared with the “first-generation” and “second-generation” systems (3-line and 2-line hybrid rice breeding system), the novel system offers a wider selection of germplasm as parental lines and is also more stable and safer for hybrid seed production.

#### Strategy for high-throughput whole-genome selection and field selection

Both extensive genotyping and high-throughput phenotyping are required throughout the process of genomic breeding. A sequence-based method has been developed to conduct genotyping and construct a genetic map for a *F*_2_ population (Huang et al. 2009). The method is approximately 20 times faster in data collection and 35 times more precise in recombination breakpoint determination compared with the conventional PCR-based method. And genotyping arrays, which integrate abundant variant information identified from diverse collections, are provided for the rapid and accurate genotyping of rice hybrids (Al-Tamimi et al. 2016; McCouch et al. 2016; Daware et al. 2023; Zhang et al. 2023). High-throughput phenotyping platforms, including greenhouse-based high-throughput rice phenotyping facility, tractor-mounted multispectral ultrasonic and reflectance sensors, gantry platforms, and unmanned aerial vehicles, are applied to collect phenotypic data of important agronomical traits throughout the whole growing period (Wing et al. 2018). Remarkable strides have been achieved in the last decade, and the field continues to experience rapid advancements.

## Concluding remarks

The problem of heterosis is in essence the problem of the inheritance of quantitative characters. Heterosis is regulated by multiple dosage-sensitive genes, and different sets of genes are involved in shaping heterosis across various traits, breeding stages, and genetic backgrounds. Although the genetic composition of heterosis is complex, several common genetic characteristics have been revealed for crop heterosis, and they are trait-specific, quantifiable, and context-dependent. These genetic characteristics provide some insight into the future genetic elucidation and practical application of heterosis. Based on the quantifiable characteristic, the contributions of different genetic components (dominance, overdominance, and epistasis) need to be quantitatively evaluated by phenotypic variation explained and other genetic effects. Given the trait-specific characteristic, the genetic compositions of heterosis for diverse traits need to be dissected, respectively, and genetic models for different traits could aid in devising trait-specific breeding strategies. For example, most loci controlling grain quality–related traits in rice hybrids represent negative dominance effects; thus, collaborative improvement in both parental lines is advocated for grain quality improvement. Considering the context-dependent characteristic, the common rules for heterosis need to be summarized based on multiple hybrid combinations with heterosis.

Ongoing advancements in modern intelligent breeding of rice hybrids include: (i) extensive collection of comprehensive data for rice germplasms, encompassing multi-omics data. The dataset is significant to construct a core panel of germplasm and enlarge the gene pool; (ii) development of high-efficiency methods for selecting founder lines, including RiceNavi platform and GS-based methods; (iii) advancement of rapid and precise strategies for genetic improvement with or without the target loci information, including the marker-assisted selection-based rational design, the restorer improvement strategy, BBSI, and CRISPR; and (iv) development of high-throughput genotyping techniques and a phenotyping platform. As our understanding of the genetic basis of heterosis deepens, along with the expansion of gene pools and the advancement of auxiliary breeding tools and strategies, the full potential of rice heterosis is expected to be unlocked.

## Data Availability

The data supporting the findings of this study are all available in the article.
